# Duration, but not intensity cues, facilitate synchronization to complex rhythms

**DOI:** 10.3758/s13414-026-03316-6

**Published:** 2026-07-29

**Authors:** Andrea Cecilia Chavez, Juan Manuel Toro

**Affiliations:** 1 Center for Brain and Cognition, Universitat Pompeu Fabra, Carrer Ramon Trias Fargas 25-27, 08005 Barcelona, Spain; 2 Catalan Institution for Research and Advanced Studies (ICREA), Passeig Lluis Companys, 23, 08010 Barcelona, Spain

**Keywords:** Rhythm, Synchronization, Iambic-Trochaic Law, Meter, Music

## Abstract

Rhythm emerges from the temporal patterning of sounds and the expectations that shape how we hear them. Rather than passively listening to incoming events, listeners organize timing information through internal predictions and external acoustic cues. In speech, louder sounds tend to mark the start of a group, whereas longer sounds mark its end, a pattern described by the Iambic–Trochaic Law (ITL) that supports segmentation and word learning. We asked whether this perceptual grouping principle in speech could also facilitate synchronization to musical rhythms. Thirty-six adults with minimal musical training tapped along to tone sequences in simple (4/4) or complex (7/8) meters, each presented with neutral, ITL-aligned, or reverse (ITL-reversed) contours. Synchronization accuracy showed no contour effects for simple meter, but contour effects emerged in complex meter, where reverse contours produced the most accurate tapping. Error analyses indicated that prosodic contours encouraged cautious timing relative to reverse, whereas reverse contours yielded more consistent beat tracking with fewer missed taps and fewer extra taps. These findings suggest that a long initial note provided an attentional anchor that stabilized timing, and that under the conditions tested here, duration cues yielded larger and more reliable synchronization benefits than intensity cues*.* Thus, intensity and duration cues may guide musical timing through distinct, attention-based mechanisms.

**Supplementary Information:**

The online version contains supplementary material available at 10.3758/s13414-026-03316-6.

## Introduction

When we listen to a rhythmic sequence, we do not simply react to sounds as they come, we actively organize them. This organization relies on both internal expectations and external acoustic cues. We construct mental patterns, such as a steady beat or a metrical grid, that help us predict when the next sound will occur (Patel, 2007). At the same time, we also use as predictive anchors some of the acoustic properties of the sounds themselves, including their intensity and duration. These two sources of information, structural and acoustic, interact to shape how we perceive rhythmic patterns across different domains, including music and speech (e.g., Fiveash et al., 2021). In the present study, we explore whether acoustic cues that induce grouping in speech and facilitate word recognition and memory might also facilitate rhythm synchronization over musical stimuli.

In spoken language, using acoustic cues as grouping anchors serves an important functional role. For example, louder sounds are typically heard as initiating a group (trochees), while longer sounds tend to mark the end of the group (iambs; Hayes, 1995) in what has been called the Iambic-Trochaic Law (ITL). When speech follows ITL-aligned prosodic patterns, listeners are better at segmenting it into words (Bion et al., 2011), identifying its internal structure (Langus et al., 2011), and learning novel word-forms (Shukla et al., 2011). These findings suggest that acoustic contours shaped by intensity and duration might improve learning by putatively making the auditory input easier to parse. Importantly, while the grouping principles linked to the ITL have been extensively studied in linguistic contexts, they have also been observed in response to nonspeech sounds, including sequences of tones (Iversen et al., 2008; Trainor & Adams, 2000). This suggests that ITL-based grouping reflects a general auditory strategy, not one limited to language.

However, there is not enough evidence about whether processing benefits similar to the ones observed over speech can be observed in other domains, such as music. Evidence suggests that speech and music rely on similar timing systems. Hausen et al. (2013) had adults complete music perception tasks and a speech prosody task and found that individuals who performed better on a speech prosody task also tended to perform better at rhythm perception in music, even after controlling for musical training, working memory, and pitch perception. Similar patterns emerge in neural measures. Electroencephalographic studies show that brain activity locks to rhythmic patterns in both music and speech. In a direct comparison, Harding et al. (2018) had 28 adults listen to melodies and sentences with matched temporal structure. The authors observed robust phase-locking to the rhythms in both domains. More recently, te Rietmolen et al. (2024) registered intracranial EEG from epilepsy patients while they listened to natural speech and music and found that both engaged overlapping cortical networks that tracked temporal structure at rates relevant to each domain: syllabic rhythms in speech (~4–8 Hz) and beat-based rhythms in music (~1–3Hz). These studies add to a growing literature suggesting that rhythmic processing in both domains relies on shared neural timing systems (e.g., Patel, 2007), raising the question of how these systems connect to motor circuits that support prediction and action.

In the speech domain, Kotz and Schwartze (2010) proposed a framework in which a subcortico–cortical timing network linking auditory and motor systems supports temporal prediction in speech perception. fMRI work in music provides converging evidence for such auditory-motor coupling: Grahn and Brett (2007) found stronger basal ganglia and premotor activity when participants listened to metrical compared with nonmetrical rhythms, and Chen and collaborators (2008) observed that these same regions, along with the supplementary motor area (SMA) and cerebellum, were recruited not only during tapping but also when participants imagined tapping, even in the absence of overt movement. Thus, mechanisms involved in rhythm perception across domains not only involve active timing processes but also generate predictive signals that motor systems can draw on to prepare movements. This opens the possibility that ITL-aligned contours that have been shown to facilitate grouping in speech, may also enhance the performance of the predictive timing mechanisms needed to synchronize movement in musical tasks.

The ITL predicts that prosodic contours should aid grouping and thereby prediction. However, an alternative possibility, grounded in Dynamic Attending Theory, is that what matters is not grouping per se, but the temporal placement of a salient cue relative to the metrical onset. These two accounts make different predictions: if ITL-based grouping drives synchronization, prosodic contours should outperform reverse ones; if onset salience drives it, the reverse should be observed. The present design, by including both ITL-aligned and reversed contours, allows us to adjudicate between these accounts. In this study, we ask whether ITL-aligned or onset-salient duration cues better facilitate synchronization to rhythmic sequences. We presented participants with auditory sequences featuring either a simple 4/4 or complex 7/8 meter, each with one of three contours: prosodic (ITL-aligned), reverse (ITL-reversed), or neutral. We then measured synchronization using Victor–Purpura distance, which quantifies how closely each tap matched the expected timing of the sounds (Victor & Purpura, 1996).

## Methods

### Participants

Thirty-eight participants (ages 19–35 years; *M*_age_ = 25.5 years, *SD* = 5.0; 24 women) took part in the experiment. They were recruited via email from the Center for Brain and Cognition’s participants database. Eligibility criteria included normal hearing and less than 2 years of formal musical training. Participants with minimal musical training were recruited to minimize the confound of domain-specific expertise in rhythm processing. Musicians’ superior synchronization performance (e.g., Repp, 2005) and their differential sensitivity to metrical accents (Bouwer et al., 2018) could mask or amplify contour effects, making it difficult to identify more general, domain-flexible mechanisms. By testing nonmusicians, we aimed to assess whether prosodic cues support synchronization through domain-general perceptual and attentional mechanisms rather than learned musical schema.

Most participants (29/38) listed Spanish and/or Catalan as their main language(s) and were predominantly right-handed. Participants gave informed consent and were compensated with 5€ for their participation. Data from one participant were discarded due to programming error and another for not meeting the eligibility criteria.

### Apparatus

Auditory stimuli were presented in a sound-attenuated booth through a pair of Logitech Z130 speakers, positioned approximately 60 cm from the participant, alongside a Logitech ThinkCentre M920t desktop computer with an Omen by HP 25 24.5-in. LED monitor at the same distance. Tapping responses were recorded via a Kensington ValuKeyboard’s down arrow key. Stimuli were delivered and responses collected using PsychoPy (Version 2024.2.1post4).

### Stimuli and conditions

**Fig. 1:**
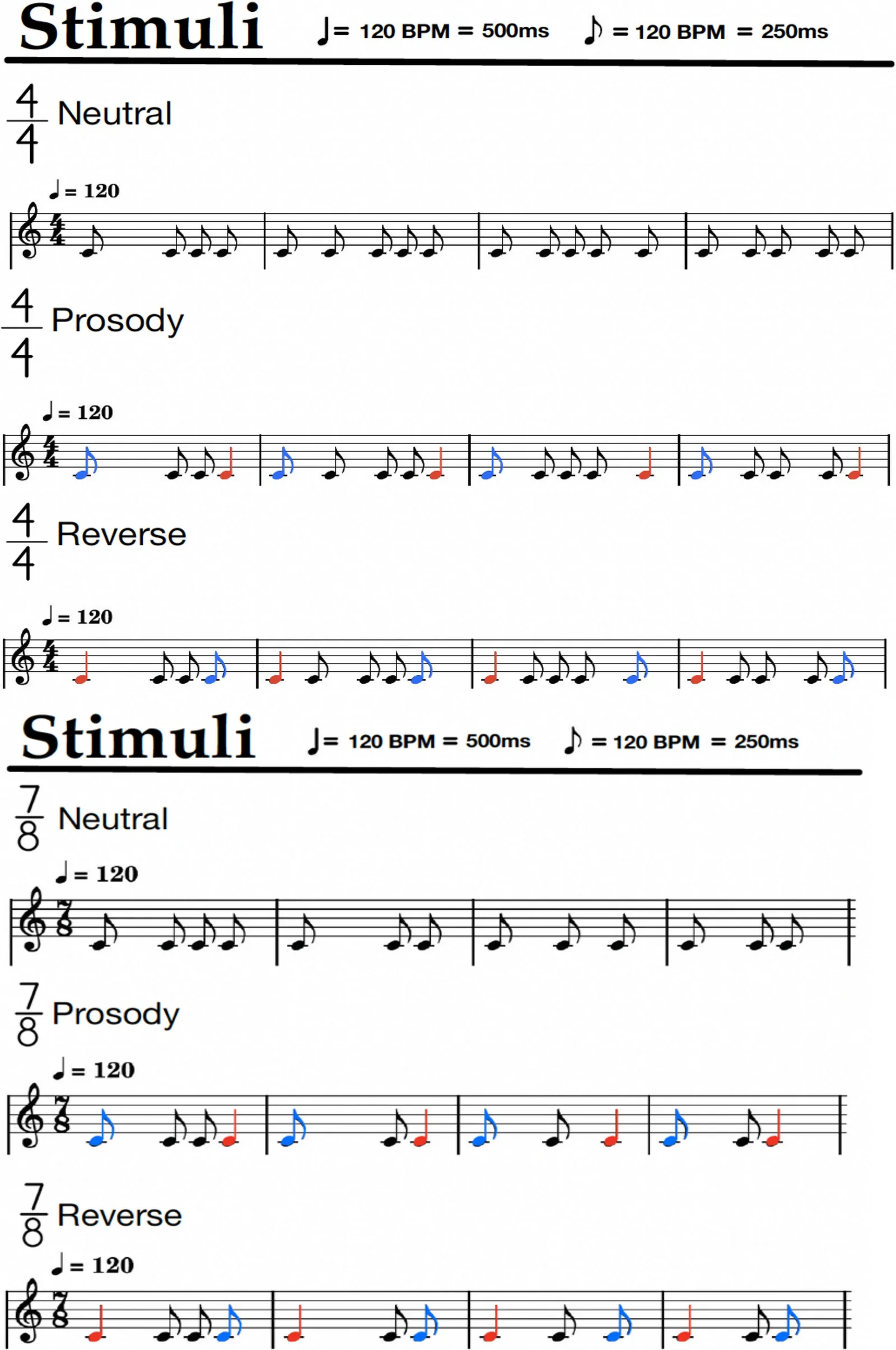
Rhythmic Stimuli by meter and contour type. Visualizations for the 4/4 (left panel) and 7/8 (right panel) meters across three contour types: neutral, prosody, and reverse. Each row shows note onsets over four measures which are repeated four times in each stimulus for a total of 16 measures. In the prosody condition, group-initial notes are emphasized via increased intensity and group-final notes via extended duration, aligning with the ITL. The reverse condition violates ITL by reversing these cues. Neutral notes had uniform duration and intensity (250 ms, ≈ 70 dB). Louder notes (colored blue) were increased by ≈ +8 dB, and longer notes (colored red) were extended to 500 ms. (Color figure online)

Stimuli were rhythmic tone sequences created in MuseScore (Version 4.2.1) and edited in Audacity (Version 3.6.3). Each sequence comprised four distinct rhythmic patterns, each repeated four times to form a 16-measure stimulus (see Fig. 1). All tones had a C4 pitch (261.63 Hz). To create a more natural and perceptually challenging rhythm, interonset intervals (IOIs) ranged between approximately 250 and 1,000 ms, while the total duration of each measure was fixed at 2,000 ms for the simple meter and 1,750 ms for the complex meter. These durations reflect the number of intervals per measure (eight in the simple and seven in the complex sequences). All sequences were played at a tempo of 120 BPM.

Each sequence was implemented following three contour conditions: neutral, prosodic, and reverse. In the neutral condition, all tones were 250-ms long and played at ~70 dB. In the prosodic condition, the first tone of each measure was louder (+8 dB, ~78 dB) and the final tone was lengthened to 500 ms. In the reverse condition, this contour was inverted: the first tone lasted 500 ms and the final tone was +8 dB louder than the others. The +8 dB increase is within the range of intensity manipulations used in previous ITL research, where prosodic grouping effects have been observed (Bhatara et al., 2015; Hay & Diehl, 2007). In total, there were six sequences, three contour conditions for each of the two meters, and each participant heard all six combinations in random order.

### Design

The study used a 2 × 3 within-subjects design crossing meter type (simple vs. complex) with contour type (neutral, prosody, reverse), resulting in six conditions. Each participant completed four trials per condition for a total of 24 trials. Trial order was randomized across participants.

### Procedure

Participants sat in a sound-attenuated booth in front of a desktop computer with a keyboard and speakers. They first completed a short familiarization phase with three practice trials guided by the experimenter, followed by 24 experimental trials. Each trial included two phases. In the listening phase, participants heard the first half of the stimulus (8 of 16 measures) and were instructed to remain still. This was done to prevent overt movement from supporting rhythm perception or encoding, as motor activity has been shown to enhance temporal prediction and entrainment (Phillips-Silver & Trainor, 2007). In the response phase, participants heard the full 16-measure sequence and were instructed to press the down arrow key in synchrony with the rhythm.

## Results

Synchronization performance was measured using the Victor–Purpura (VP) distance, a method that compares the timing of each tap to the target rhythm (Victor & Purpura, 1996). It calculates the “effort” needed to turn the participant’s tapping into the correct pattern, based on how much you would need to shift, delete, or insert taps. To decide how costly a shift is, we used a scale based on the rhythm’s average timing. Specifically, we set the shift cost to: $Cost\left(shift\right)=q \times {\boldsymbol{\updelta}}$**,** where **δ** is the time difference (in seconds) between a tap and a note onset, and *q* is a cost parameter defined as:

$$q=\frac{1}{\overline{IOI} },$$

with IOI being the mean interonset interval of the stimulus notes.

This means that shifting a tap by the average IOI between notes costs the same as skipping the tap entirely and assuming it was a missed beat. In other words, small timing errors are treated as minor adjustments, while larger mismatches are penalized more heavily. For example, a tap that is 50 ms off the target might be treated as a minor misalignment, while a tap 500 ms off would be considered a major error. Lower VP scores indicate closer alignment between the participant's taps and the stimulus sequence. Importantly, simple meter (4/4) sequences have 76 notes while those of complex meter (7/8) have 52, giving more chances for errors in the simple meter condition. Matching note counts would distort rhythmic structure, so analyses were conducted separately for each meter. Across all analyses reported below, pairwise comparisons following a significant omnibus test were corrected for multiple comparisons using the Bonferroni method (three contour contrasts per family, corrected α =.0167); corrected *p* values are reported as *p_corr* alongside uncorrected *p_unc* values.

### Participant-level averages

**Fig. 2:**
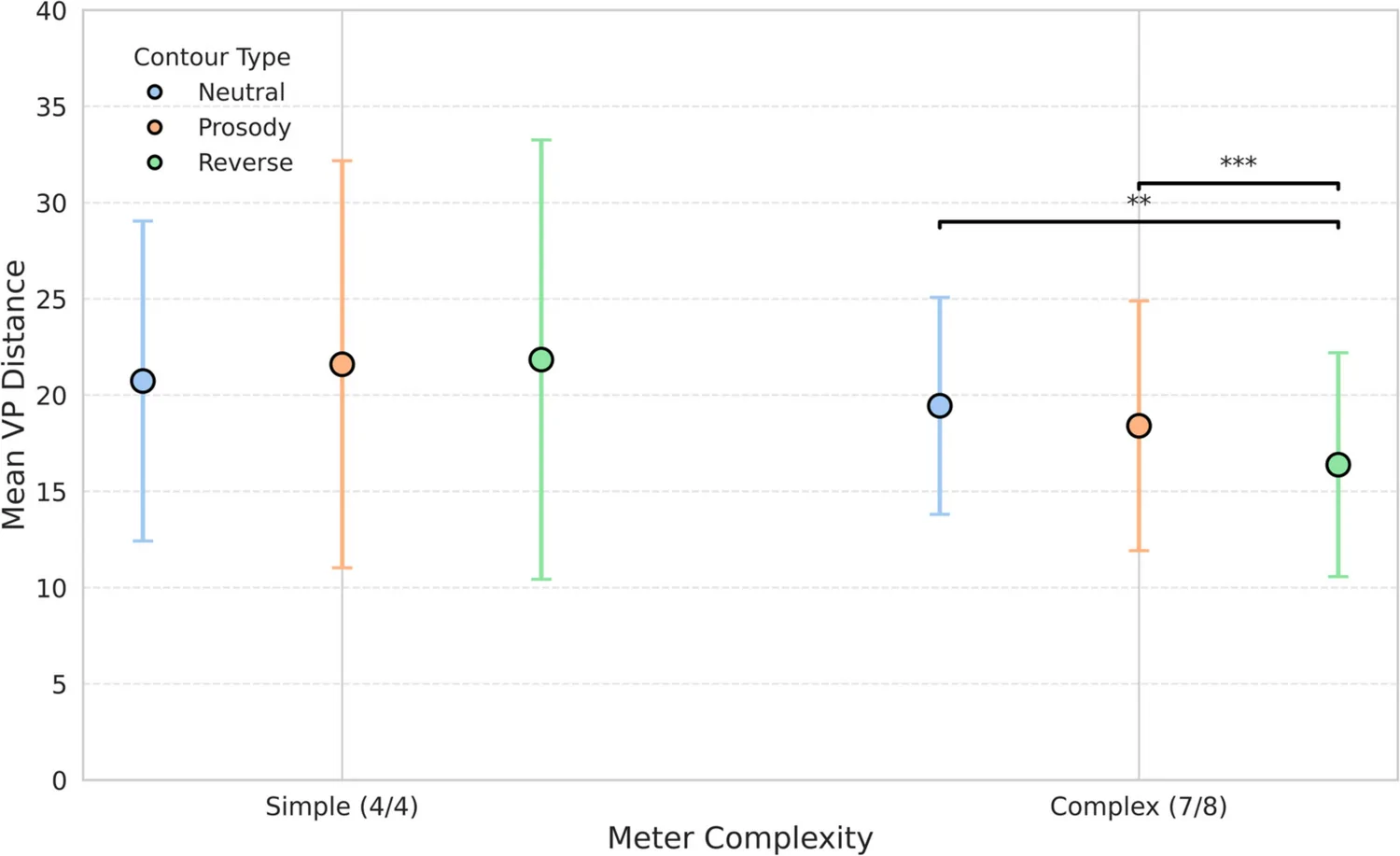
Mean Victor–Purpura (VP) distance by meter complexity and contour type (aggregated across trials). Dots show the average VP distance for each contour condition (neutral, prosody, reverse) in simple (4/4) and complex (7/8) meters. Error bars represent standard deviations across trials. Lower values indicate better synchronization. In complex (7/8) meter, reverse contour significantly reduced VP distance relative to neutral, with reverse also outperforming prosody. No significant differences were observed between contours in simple meter. Asterisks denote significance levels (*p <.05, **p <.01, ***p <.001). (Color figure online)

Figure 2 displays participant-level means where each data point reflects the average across trials per participant and condition. To assess whether contour had an overall effect within each meter, a one-way repeated-measures analysis of variance (ANOVA) was conducted for each meter with three contour conditions (neutral, prosody, reverse).

For the sequences following a simple meter, the main effect of contour was not significant, *F*(2, 70) = 1.38, *p* =.26, η^2^ =.007, indicating no reliable differences among conditions. This pattern was confirmed by the pairwise comparisons, none of which reached significance: neutral (*M* = 23.84, *SD* = 5.38) vs. prosody (*M* = 25.44, *SD* = 8.87), *t*(35) = −1.35, *p_unc* =.186, *p_corr =*.559*, d* = −0.22, 95% CI [−4.02, 0.81]; neutral vs. reverse (*M* = 25.01, *SD* = 9.00), *t*(35) = −1.04, *p_unc* =.305, *p_corr =*.913, *d* = −0.17, 95% CI [−3.45, 1.11]; and prosody vs. reverse, *t*(35) = 0.78, *p_unc* =.442, *p_corr =* 1.00, *d* = 0.13, 95% CI [−0.70, 1.57].

In contrast, for the sequences following a complex (7/8) meter, the ANOVA revealed a significant main effect of contour, *F*(2, 70) = 14.19, *p* <.001, η^2^ =.083. Pairwise comparisons showed that synchronization performance under prosody (*M* = 20.27, *SD* = 5.33) was not significantly different from neutral (*M* = 21.66, *SD* = 4.05), *t*(35) = 1.96, *p_unc* =.058, *p_corr =*.173*, d* = 0.33, 95% CI [−0.05, 2.82]. Synchronization was markedly better under reverse (*M* = 18.12, *SD* = 5.19) than both neutral, *t*(35) = 4.49, *p_unc* = <.001, *p_corr* <.001, *d* = 0.75, 95% CI [1.95, 5.13], and prosody contours, *t*(35) = 4.51, *p_unc* <.001, *p_corr* <.001, *d* = 0.75, 95% CI [1.18, 3.12]. Together, these analyses show that contour manipulations did not influence synchronization in simple meter but significantly improved performance in complex meter, with reverse contours producing the strongest benefit.

### Validated trials

To ensure the observed effects were not driven by noisy data, we applied a validation screen, excluding trials where tap count deviated by more than 15% from the number of note onsets. For every trial, we computed the absolute tap–onset mismatch as:

$$\mathrm{p}\mathrm{c}\mathrm{t}\mathrm{m}\mathrm{i}\mathrm{s}\mathrm{m}\mathrm{a}\mathrm{t}\mathrm{c}\mathrm{h}= \frac{\left|{n}_{taps}-{n}_{onsets}\right|}{{n}_{onsets}}.$$

**Fig. 3:**
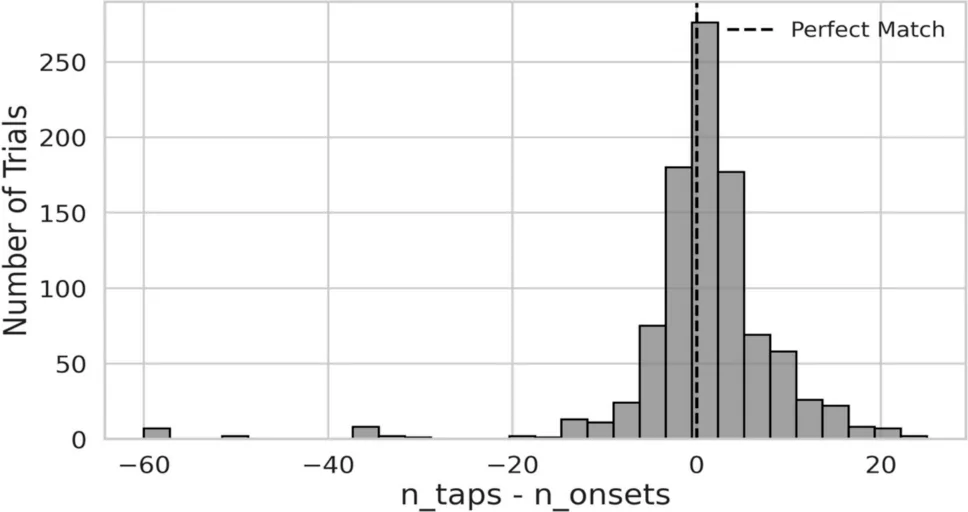
Distribution of tap–onset count differences across trials. Histogram shows the difference between the number of taps and the number of stimulus onsets for each trial. The dashed line at zero indicates a perfect match. Most trials cluster around zero, indicating close alignment between tap and onset counts. Trials were retained for analysis if this mismatch was within ±15% of the expected number of onsets, forming the validated trial set. Outliers far from zero were excluded

Trials were retained when this mismatch did not exceed 15% of the notes. The 15% filter excluded 148 out of 971 trials (15.2%), leaving 823 valid trials (84.8%) contributed by all 36 participants (see Fig. 3). Within the retained set, 66 of the 72 simple/complex participant × meter cells contained data for all three contour conditions; rows with missing cells were omitted from paired analyses, yielding samples of 32–35 participants per contrast. We then further restricted our analysis to participants who had valid data in all three contour conditions within each meter, yielding a fully balanced sample of 34 in simple meter and 32 in complex.

### Individual differences in synchronization skill

Prior work has shown that synchronization performance varies widely across individuals, with some participants consistently showing tighter alignment to the target rhythm and others exhibiting greater timing variability (e.g., Repp, 2005). To account for this variability, we screened participants based on their mean asynchrony in the first practice trial. This trial featured a 9-s-long isochronous sequence with the C4 tone, offering a neutral and controlled context for measuring baseline synchronization skills. To eliminate false starts and ensure consistency, the first four note onsets were trimmed from the analysis.

Participants with absolute mean asynchrony >150 ms were labeled as poor synchronizers. This threshold reflects a conservative cutoff for simple isochronous synchronization tasks. Only two participants (5.6%) met this criterion, so no formal statistical comparison between skills groups was conducted.

### Error type analysis

To better understand how prosodic cues influenced tapping behavior, we examined not only overall alignment captured by VP distance, but also the types of errors participants made. While VP distance summarizes overall alignment, it does not distinguish between different kinds of failures such as tapping too few or too many times. Therefore, we analyzed extra taps (more taps than expected) and missed taps (fewer taps than expected) as complementary metrics.

**Fig. 4:**
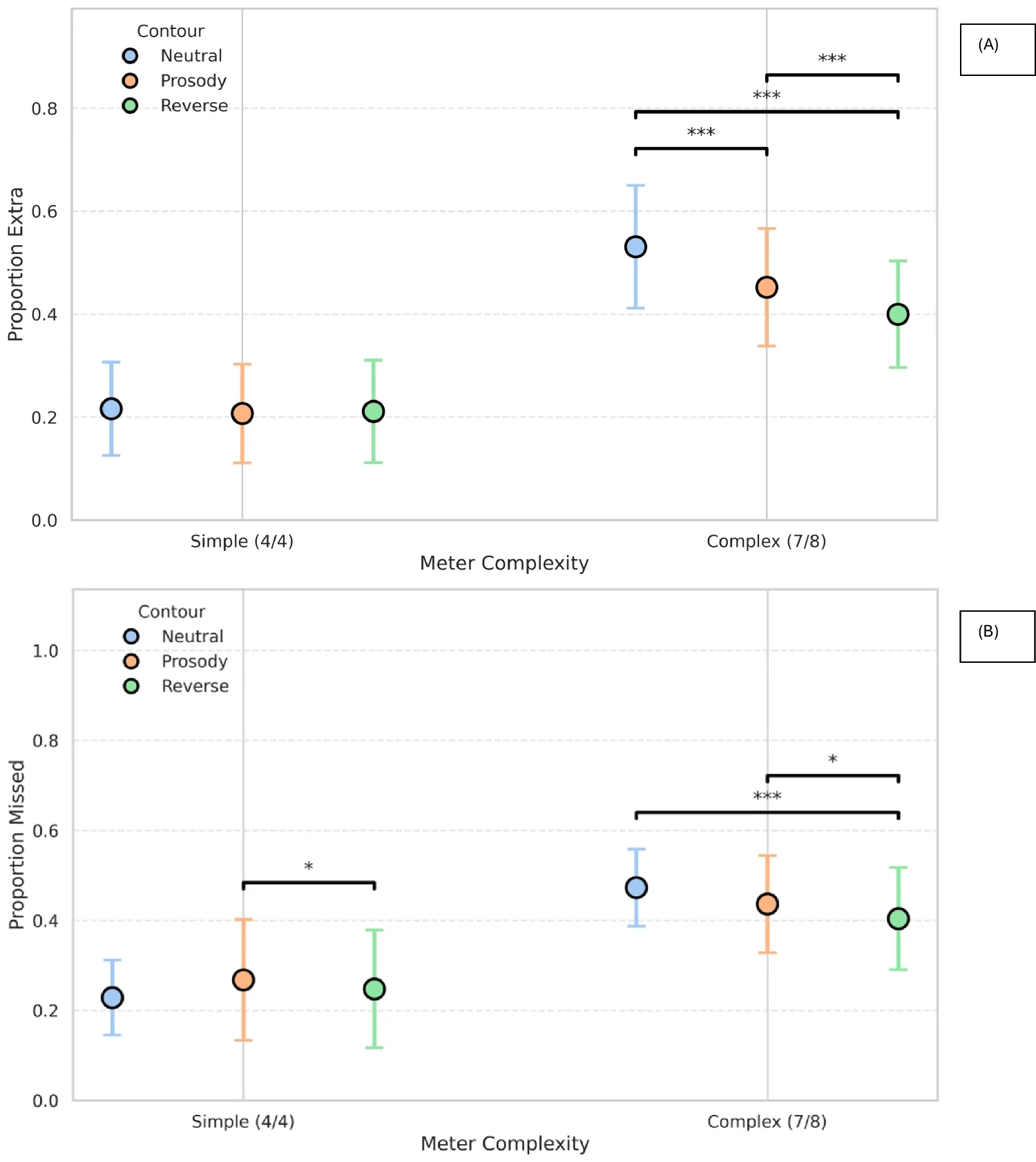
Tapping error rates by meter and contour type. **(A)** Proportion of extra taps*.* In complex (7/8) meter, both prosodic and reverse contours significantly reduce extra taps compared to the neutral baseline, with reverse contour showing the largest reduction. No significant differences are observed in simple (4/4) meter. **(B)** Proportion of missed taps. In simple (4/4) meter, prosody showed a trend toward higher missed taps than neutral that did not survive correction for multiple comparisons, while the neutral versus reverse contrast was nonsignificant at either level; prosody showed significantly more missed taps than reverse, even after correction. In complex (7/8) meter, reverse significantly reduced missed taps relative to neutral after correction, while prosody showed only a trend versus neutral that did not survive correction; prosody significantly exceeded reverse in missed taps even after correction. Error bars reflect within-subject standard errors and asterisks denote significance levels after Bonferroni correction (*p_corr <.05, **p_corr <.01, ***p_corr <.001). (Color figure online)

Extra taps showed a distinct pattern (see Fig. 4A). In the sequences following a simple (4/4) meter, no contour comparisons reached significance (all *p_unc* ≥.35, *p_corr =* 1.00), indicating that prosodic cues had minimal influence on overtapping. In contrast, in complex (7/8) meter, extra taps decreased under prosodic, *t*(35) = 4.43, *p_unc* <.001, *p_corr* <.001, *d*_z_ = 0.74, 95% CI [0.04, 0.11], and reverse contours, *t*(35) = 7.99, *p_unc* <.001, *p_corr* <.001, *d*_z_ = 1.33, 95% CI [0.10, 0.16] relative to neutral. Reverse also showed fewer extra taps than prosody, t(35) = 4.65 *p_unc* <.001, *p_corr* <.001, *d*_z_ = 0.78, 95% CI [0.03, 0.08]. These results suggest that both contour types helped participants avoid overtapping in complex meter, with the reverse contour producing the strongest reduction.

As shown in Fig. 4B, missed taps in the simple meter (4/4) condition showed a trend toward being higher under prosody compared to neutral, though this did not survive correction for multiple comparisons (prosody vs. neutral), *t*(35) = −2.36, *p_unc* =.024, *p_corr* =.072, *d*_z_ = −0.39, 95% CI [−0.07, −0.01], while the neutral vs. reverse contrast was non-significant, *t*(35) = −1.18, *p_unc* =.247, *p_corr* =.744, *d*_z_ = −0.20, 95% CI [−0.05, 0.01]. Interestingly, prosody also led to significantly more missed taps than reverse contours, even after correction, *t*(35) = 2.73, *p_unc* =.010, *p_corr* =.030, *d*_z_ = 0.46, 95% CI [0.01, 0.03], suggesting that cue salience may have induced uncertainty or hesitation.

In the complex (7/8) meter condition, reverse significantly reduced missed taps relative to neutral, even after correction, *t*(35) = 4.14, *p_unc* <.001, *p_corr* <.001, *d*_z_ = 0.69, 95% CI [0.04, 0.10], while prosody showed only a trend towards more missed taps than neutral that did not survive correction (*t*(35) = 2.43, *p_unc* =.020, *p_corr* =.061, *d*_*z*_ = 0.41, 95% CI [0.01, 0.07]). Prosody also significantly exceeded reverse in missed taps even after correction, *t*(35) = 2.58, *p_unc* =.014, *p_corr* =.042, *d*_z_ = 0.43, 95% CI [0.01, 0.06], reinforcing the advantage of the reverse contour in aiding tap timing.

We also looked at the moment within the beat that participants chose to tap. More specifically, whether they tended to tap slightly before or after each note onset. In simple meter, this pattern did not differ between contour types, *F*(2,70) = 2.56, *p* =.085. In complex meter, however, a difference emerged, *F*(2,70) = 5.48, *p* =.006, ηg^2^ =.050: participants tapped early less often when following the reverse contour compared with both the neutral, *t*(35) = 3.01, *p_*unc =.005, *p_corr =*.015, *d* = 0.50, and prosody conditions, *t*(35) = 2.69, *p_*unc =.011, *p_corr* =.033, *d* = 0.45. This suggests that the reverse contour not only helped participants tap more consistently but also encouraged them to wait slightly longer before committing to each tap, applying a more cautious, well-timed response style.

To check whether contour type shifted the overall timing of taps, we calculated mean signed asynchrony. This is the average time difference between each tap and its nearest note onset, where negative values mean tapping early. Across all conditions, participants tapped slightly late rather than early (by around 22–27 ms), and this did not vary by contour in either meter, simple: *F*(2,70) = 2.36, *p* =.10; complex: *F*(2,70) = 0.85, *p* =.43. So contour type did not shift tapping earlier or later overall. It specifically reduced the tendency to tap early in complex meter, a subtler effect on response consistency rather than timing in general.

### Learning across trials

**Fig. 5:**
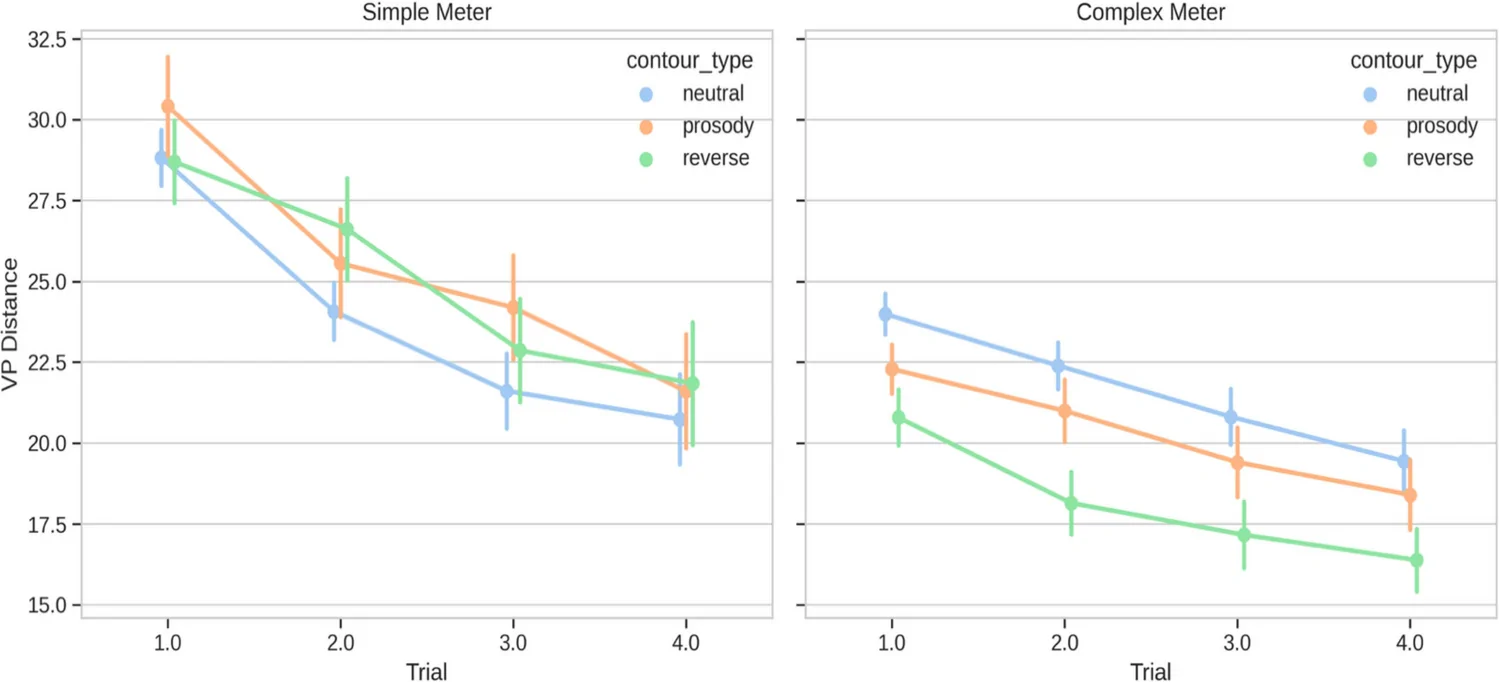
Change in VP distance from Trial 1 to Trial 4 by meter and contour. Each panel shows average VP distance (lower = better alignment) across four trials for each contour. Error bars represent standard error of the mean. In both meters, VP distance decreased across trials, indicating learning. However, learning rates did not differ by contour type, suggesting that prosodic and reverse cues did not facilitate learning beyond general practice effects. (Color figure online)

We then assessed whether synchronization improved with repeated exposure, and whether contour cues modulated such learning. As shown in Fig. 5, VP distance significantly decreased across trials in both meters, indicating that participants improved with practice. In simple (4/4) meter, the effect of trial was strong (β = −2.69, *p* <.001), and neither contour nor the trial × contour interaction were significant, indicating that the benefit of practice was uniform across all contour conditions. In complex (7/8) meter, synchronization also improved across trials (β = −1.52, *p* <.001). Reverse contour significantly lowered overall VP distance relative to neutral (β = −3.80, *p* <.001). However, no contour × trial interactions were significant for any contrast, showing that contour cues did not accelerate learning: participants improved at the same rate across trials. All other contour contrasts were likewise non-significant.

## Discussion

In the present study, we tested whether intensity and duration changes at the beginning and end of rhythmic sequences facilitate synchronization. The results showed that the effect of these cues depended on rhythmic complexity. In sequences following a simple meter (4/4), synchronization did not significantly differ across contours. In sequences following a complex meter (7/8), by contrast, both prosodic and reverse contours improved performance relative to a neutral contour, with reverse contours triggering the largest benefit. Error analyses revealed complementary patterns: reverse contours reduced both missed and extra taps, while prosody contours were linked to more misses. Synchronization improved with practice across trials, but contour cues did not alter the rate of learning.

The ITL proposes that louder sounds mark group beginnings and longer sounds mark group endings (Hayes, 1995). If these grouping rules helped listeners to parse rhythmic sequences more efficiently, one would expect that prosodic contours should trigger a better synchronization to the rhythm. Instead, reverse contours outperformed prosodic ones. It thus appears that, under the present experimental conditions, what facilitates synchronization is not boundary-marking per se but the temporal placement of a salient duration cue at the metrical onset.

The ITL describes a reliable pattern in how listeners use acoustic cues to segment speech, but the present findings suggest that what drives perceptual benefits in music may not be the grouping rule itself, but the attentional salience of duration at structurally prominent positions. In speech, final lengthening happens to coincide with a boundary, making it useful for segmentation. In music synchronization, the present results suggest that what matters is not boundary marking but forward anchoring: a salient cue at the metrical onset orients attention toward what comes next. That this cue violates ITL predictions is therefore not surprising, as it reflects the fact that the temporal demands of synchronization differ from those of segmentation, and that duration salience serves prediction best when placed at the onset rather than the boundary.

This outcome aligns more closely with Dynamic Attending Theory (DAT; Large & Jones, 1999; Large et al., 2023), which emphasizes that attention is guided by the salience and timing of events rather than fixed grouping templates. From this perspective, the long initial note in the reverse condition acted as a reliable anchor that oriented participants to the rhythm and supported the predictions of what followed in the sequence. This might help to explain why contour effects appeared during the synchronization to sequences following complex but not to sequences following simple meters.

In the rhythmic sequences following a simple 4/4 meter, the familiar, regular structure provided a stable framework for internal timing, leaving little room for external cues to improve performance. Simple-duple meters such as 4/4 are prevalent in Western repertoires (London, 2004), and adults exposed to these repertoires show heightened sensitivity to their structure (Hannon & Trehub, 2005). This familiarity likely engaged stable internal timing mechanisms supported by basal ganglia and pre-SMA/SMA networks (Grahn & Brett, 2007). Strong endogenous entrainment at the beat (Nozaradan et al., 2016) may have maintained accurate synchronization across all contours, explaining the absence of contour effects in synchronization to sequences following this meter.

In contrast, in the rhythmic sequences following a more complex 7/8 meter, the irregular grouping made it more difficult to sustain timing internally, increasing reliance on external anchors. Boundaries in a sequence naturally attract attention (Jones & Boltz, 1989), and long notes act as temporal accents that strengthen this effect (Jones, 1987). When a long note occurred at the beginning of a measure, it provided a salient reference point: attention was drawn to the onset, and internal attending rhythms were realigned to the external pulse (Large & Jones, 1999). This alignment enhanced sensitivity to upcoming events and stabilized entrainment across the sequence, giving listeners a temporal framework for prediction. In this way, a sequence-initial lengthening supported forward-looking temporal expectations, which is particularly advantageous in metrically complex contexts where internal models are less robust.

This account is consistent with evidence that temporal accents on metrically strong positions facilitate beat perception (Bouwer et al., 2018), though this effect is often larger in musicians. The present observation of similar benefits in nonmusicians suggests that such cues may also recruit domain-general mechanisms of attentional alignment and temporal prediction. By contrast, a final-note lengthening primarily reinforced phrase closure (Jones & Boltz, 1989), supporting retrospective segmentation but providing little guidance for anticipating the next beat.

The error analyses add further insight into how cues shaped synchronization. Prosodic contours showed a numerical trend toward more missed taps than neutral contours in both meters, though this did not survive correction for multiple comparisons; prosodic contours did, however, lead to significantly more missed taps than reverse contours even after correction. This pattern suggests that although an intensity cue marked the beginning of each measure, it may have been less effective than a salient duration cue at anchoring temporal predictions, potentially leaving participants more hesitant and resulting in more missed taps than the reverse condition. Reverse contours, by contrast, promoted more consistent engagement with the rhythm, committing to more beats overall and producing fewer misses and fewer extra taps. Notably, reverse contours also produced fewer early responses in complex meter, suggesting that the longer onset note did not simply trigger impulsive tapping but instead supported more stable, beat-locked responding. These contrasting patterns suggest that the type of acoustic cue at the measure onset shaped not just the accuracy of synchronization but also the style of temporal engagement, with duration at the onset supporting more committed, sustained tracking of the rhythm than intensity, consistent with accounts of temporal accents shaping attentional engagement (Jones, 1987).

Further, we considered whether the reverse contour benefit might reflect a purely mechanical advantage rather than an attentional one. Because the lengthened onset note makes the first sound in each measure last twice as long (500 ms vs. 250 ms), participants may have simply had more time to prepare and execute their tap, rather than benefiting from an attentional anchor. If that were the case, we would expect the overall timing of responses to shift earlier under the reverse contour, as participants exploit the wider window to tap sooner. However, mean signed asynchrony did not differ across contour conditions in either meter, indicating that the lengthened onset note did not shift the overall timing of responses earlier. More tellingly, reverse contours specifically reduced the proportion of early taps in complex meter (vs. neutral: *d* = 0.50; vs. prosody: *d* = 0.45), without shifting mean asynchrony. This pattern, fewer early taps but no shift in overall timing, is more consistent with attentional anchoring at the metrical onset improving beat-locked precision than with a simple widening of the response window. While these strategies reveal how cues shaped moment-to-moment behavior, the learning analysis shows how performance changed across trials. Although performance improved across trials in both meters, contour cues did not accelerate this learning. This indicates that salient anchors were most effective at the beginning of entrainment, when the structure was still unfamiliar, while longer-term improvements came from practice and general adaptation. That is, their effect was limited to initial alignment rather than ongoing learning.

These results extend previous work linking rhythm in music and speech (Harding et al., 2018; Patel, 2007; te Rietmolen et al., 2024), with a focus on whether prosodic cues support synchronization in music as they support segmentation and word learning in speech. In language, ITL-aligned contours mark beginnings and endings, which facilitates segmentation and prediction (Bion et al., 2011; Langus et al., 2011; Shukla et al., 2011). By the same logic, if similar cues highlighted rhythmic groupings in music, they could have provided anchors for sensorimotor timing. Yet in the present study, prosodic contours did not produce the strongest synchronization benefits. Instead, performance was best supported by salient anchors, consistent with Dynamic Attending Theory.

While the ITL has often been understood as universal, cross-linguistic studies show that its effects are modulated by listeners’ native language. Hayes (1995) noted that the trochaic effect of intensity (strong–weak) is generally weaker than the iambic effect of duration (short–long), and later work confirmed that duration-based grouping is more sensitive to the prosodic structure of the listener’s native language, whereas intensity-based grouping is relatively stable but less robust (Bhatara et al., 2013; Iversen et al., 2008; Molnar et al., 2014; Yoshida et al., 2010). Languages such as English and Spanish, where shorter function words precede longer stressed content words, typically favor iambic grouping. On the other hand, languages like Japanese and Basque, which mark prominence at the start of phrases or syllables, exhibit a trochaic or mixed bias. Because most of the participants in the present study were native speakers of Spanish, a language in which the natural prosodic cues align well with the principles described by the ITL, the absence of a prosodic contour advantage cannot be attributed to language specific differences.

The present findings suggest that ITL principles, while robust in linguistic contexts, do not extend straightforwardly to synchronization. Our results suggest that rhythmic grouping cues and temporal salience may play complementary roles, with grouping aiding perception and memory, and salience guiding real-time action. In music, this division suggests that prosodic cues may be more useful for learning a rhythm than for keeping pace with it in real time. Future work can test this possibility with tasks that probe both memory and motor synchronization.

Several limitations of the present study should be noted. The current design cannot fully dissociate the perceptual and motor contributions of the lengthened onset note. Extending a tone from 250 to 500 ms introduces a duration-based accent but also alters the local execution window available for tapping. While the signed asynchrony and early-tap data argue against a pure affordance account, the two mechanisms could co-occur, and a parametric manipulation of onset duration would be needed to dissociate them. Subsequent work could also explore whether more salient intensity contrasts, or intensity manipulations matched in perceptual salience to the duration change, would produce comparable synchronization benefits.

Further studies could also test conditions where onset duration is varied more gradually, or where a silent gap of equivalent duration replaces the lengthened tone, to isolate attentional from motor contributions. Additionally, the criterion of fewer than 2 years of formal musical training may not fully capture individual differences in rhythmic experience. For example, even a single year of percussion instruction could substantially alter beat-based timing mechanisms while still meeting our inclusionary threshold (cf. Bouwer et al., 2018). Research in this area would benefit from more sensitive measures of musical engagement, such as the Goldsmiths Musical Sophistication Index (Gold-MSI; Müllensiefen et al., 2014) or rhythm-specific aptitude tests, to better characterize participant variability and assess how musical experience moderates the effects of prosodic cues on synchronization.

Taken together, the present results indicate that synchronization benefits more from early anchors that orient attention forward in time than from closing cues that emphasize what has already elapsed.

## Supplementary Information

Below is the link to the electronic supplementary material.

Supplementary file1 (TXT 28 KB)

Supplementary file2 (ZIP 37117 KB)

## Declarations

### Open practices statement

All data and materials, including trial-level tapping data and stimuli, are provided in the supplementary material. Analysis code is available upon request from the corresponding author. This study was not preregistered.
